# Turkish adaptation of Ghosting Questionnaire: validity and reliability study

**DOI:** 10.1186/s40359-025-02677-1

**Published:** 2025-04-17

**Authors:** Ayşe Ece Atalar, Nurdan Çolakoğlu, Zeynep Bağişlioğlu, Achraf Ammar, Haitham Jahrami

**Affiliations:** 1 Istanbul Medipol University, Istanbul, Türkiye; 2 Istanbul Arel University, Istanbul, Türkiye; 3 Department of Training and Movement Science, Institute of Sport Science, Johannes Gutenberg- University Mainz, Mainz, Germany; 4 Research Laboratory, Molecular Bases of Human Pathology, LR19ES13, Faculty of Medicine of Sfax, University of Sfax, Sfax, 3000 Tunisia; 5 High Institute of Sport and Physical Education of Sfax, University of Sfax, Sfax, Tunisia; 6 Department of Psychiatry, College of Medicine and Health Sciences, Arabian Gulf University, Manama, Bahrain; 7 Government Hospitals, Manama, Bahrain

**Keywords:** Ghosting, Scale adaptation, Cyberpsychology, Social media

## Abstract

**Background:**

Ghosting is a newly studied term that is mostly used for online relationships. It is described as one of the methods used for breakups. As follows, the channels of communication are sharply ended without explanation.

**Objectives:**

This study aims to examine the psychometric properties of the Ghosting Questionnaire, which was developed to measure ghosting behavior, in Turkish culture. There are limited methods for quantitative research, as it has only recently found its place in the field. For the Turkish adaptation of the scale developed by Jahrami et al. (2023), a process consisting of translation, data collection, and analysis was followed.

**Method:**

The study group consisted of 200 adults aged 18–39 years, 160 (80%) of whom were female and 40 (20%) of whom were male. The factor structure, internal consistency, and convergent validity were examined within this study. The Ghosting Questionnaire’s unidimensional structure was confirmed by CFA as part of the validity assessment. Cronbach’s alpha was used to evaluate the scale’s reliability.

**Results:**

The results of the exploratory factor analysis support the unidimensional structure as in the original scale. The confirmatory factor analysis results show that the fit indices are at acceptable levels (GFI = 0.97, CFI = 0.99, NFI = 0.97, RFI = 0.94, IFI = 0.99 and RMSEA = 0.03). Corrected item correlations ranged from 0.417 to 0.710. The Cronbach’s alpha coefficient results show that the test is highly reliable (α = 0.86).

**Conclusions:**

These findings suggest that the Turkish scale has appropriate psychometric validity and reliability and can be used to measure ghosting behavior in the Turkish sample.

## Introduction

Increasing the use of social media causes both positive and negative feedback. It is undeniable that social media enables communication tools to work faster, which opens another door to relationship development; therefore, the relationship process has changed over the last 10 years [1]. The rise of dating apps, launched almost 25 years ago, helps both women and men enter romantic and sexual relationships more easily by providing information about others before arranging a face-to-face date [2, 3]. On the other hand, there are some online-based negative effects on relationships, such as risk-taking behaviors, and cyberstalking, and some new terms, including “ghosting”, “benching”, and “breadcrumbing” [4]. These terms emerged because of the need to describe new online dating behaviors, including flirting and starting or ending a relationship [5]. This paper aims to examine ghosting behavior and adopt the “Ghost Questionnaire” developed by Jahrami et al. [6] in Turkish. This adaptation study aims to fill the gap in the literature and find a place for the ghosting concept, which has started to take place relatively recently emerged as a tool to be used in research.

### Ghosting

The word ‘ghosting’ was defined in the Urban Dictionary [7] as “When a person cuts off all communication with their friends or the person they’re dating, with zero warning or notice beforehand. You will mostly see them avoiding friends’ phone calls, social media, and avoiding them in public”. Accordingly, ghosting behavior is described as a dissolution of a relationship. This is one of the strategies used to end a relationship [8]. The main difference from any other breakup with a romantic partner or a friend is that they avoid any sort of communication, which ends up leaving without any explanation [9], and young people are open to ending relationships through online platforms and are likely to do so if it is an early stage of a relationship or a short-term one most likely started online [10, 11]. Another difference in ghosting is that the act of breakup actually happens without the one who ghosts the other on purpose. This is how breaking up is seen as cutting off the communication by the ghoster, which is unilaterally accessing other(s) provoked relationship dissolution commonly enacted through one or several technological medium(s) is what is meant by the term “ghosting.” [1]. In other words, those who engage in ghosting just vanish from their partners’ lives [12].

Breaking up through online platforms has not emerged recently. Texting has seemed to be the most frequently used one of all methods to end a relationship [13]. Previous research involving 554 participants revealed that 25.3% had ghosted and that 21.7% had previously ghosted a romantic partner once. In the same study, participants were asked to define how to expose ghosting behavior, and 87.5-95.8% of all reported not contacting/responding to texts or phone calls [8]. Another study revealed that 34.8% of participants whose relationships started via online platforms claimed to have been ghosted, and 45.4% of them ghosted others [14]. Texting, voice mailing, or connecting on SNSs (Social Network Sites) related behaviors are suggested to be least warmth and affectionate. In this case, technology-mediated breakups can equal avoidance through distant communication, in which people distance themselves from their partners either emotionally or physically [15].

### Psychological effect of ghosting behavior

As a relationship-ending strategy, ghosting is an extreme form of withdrawal or avoidance. Avoidance and withdrawal are less likely to be well-received by recipients than open confrontation strategies (such as verbal confrontation), and they are linked to higher levels of distress after the relationship ends [16, 17]. In relationships, expressing emotions is healthy, and suppressing them can significantly harm a relationship. Furthermore, while anger can exacerbate already existing relationship problems, experiencing being hurt can help them heal [18].

It has been observed that increased use of social media is associated with increased levels of being a victim of ghosting as well as mental health problems. Negative emotions related to mental health, such as sadness, hurt, anger, frustration, and disappointment are related to being a victim of ghosting [19]. Women using online dating applications were found to use ghosting more frequently than face-to-face rejection. Self-presentation, the other party’s self-presentation, and ghosting behavior before rejecting the relationship affected the level of stress. This can be considered a strategy that eliminates the possible cause of stress even before meeting face-to-face [20]. Another study of people who had experienced ghosting behavior revealed that ghosting led to worse outcomes (i.e., emotions, basic psychological needs, cognitive appraisal of separation, and aggressive tendencies) than rejection did [12]. Although it is not a verbal rejection, ignoring the other shows the behavioral output that the ghoster does not want to be in the relationship. In another study, many of the participants reported that the experience of ghostees had a detrimental effect on their self-esteem and trust in others, which is in line with psychological research showing that self-esteem can decrease when they experience rejection [21].

## Materials and methods

### Study design

The Ghosting Questionnaire was translated as recommended by Beaton et al. [22], and the final version was assessed for validity.

### Participants

Data were collected using Google Forms with all questions programmed as mandatory fields, which prevented submission of incomplete responses and ensured no missing values in the dataset. Participants were automatically excluded when the consent form was not accepted. A total of 200 individuals aged 18 to 39 completed the survey. The mean age was 25.1 years, and 80% were female.

We conducted boxplot analysis to identify potential outliers. Descriptive statistics, including means, standard deviations, skewness, and kurtosis values for all GHOST items are presented in Table 1. As shown, skewness values ranged from 0.096 to 1.228, and kurtosis values ranged from − 1.166 to 1.172, indicating some items deviated from perfect normality but remained within acceptable limits (± 2) for parametric analyses. Additionally, we conducted Mardia’s Test of Multivariate Normality, which indicated deviations from multivariate normality (Skewness = 10.479, χ²(120) = 349.295, *p* = 0.001; Kurtosis = 98.916, z = 10.574, *p* = 0.001). Despite these deviations, we proceeded with our analyses as factor analysis is generally robust to violations of normality with adequate sample sizes.

### Instruments

Ghosting Questionnaire was developed by Jahrami et al. [6]. The scale is a self-evaluation scale consisting of 5 Likert-type items that vary between “never” (1) and “always” (5) and has 8 items in total. According to the reliability analysis, Cronbach’s α, the ordinal α, and McDonald’s ω were 0.74 (95% 0.70; 0.76), 0.80 (95% 0.76; 0.82), and 0.74 (95% 0.70; 0.75), respectively. The CFA results demonstrated the statistical significance of the baseline and factor models, with χ2 (df) = 507 (28), *p* = 0.001, and χ2 (df) = 57 (20), *p* = 0.001. The fit indices showed that the results were highly fit, with RMSEA = 0.07, GFI = 0.99, and CFI = 0.92. High dynamic fit = Level 1: 90/10 SRMR = 0.03; RMSEA = 0.03; CFI = 0.98 was also demonstrated by the DFI cutoff results. Level 2: RMSEA = 0.06, CFI = 0.97, 95/5 SRMR = 0.05. Level 3: RMSEA = 0.09, CFI = 0.95, and 95/5 SRMR = 0.06.

### Ethical considerations

For the “Ghosting Questionnaire” study developed by Jahrami et al. [6], permission was obtained from the developer, who is also a co-author via e-mail. Afterward, a form for electronic informed consent was signed by each participant. The goal of the study, its private nature, and how the anonymous data were processed were all described in the consent form. Ethical approval was obtained from The Research Ethics Committee of Istanbul Arel University by the decision dated 24.05.2024 numbered 03 for study E-52857131-050.04-633120.

### Translation

The suggested steps of cross-cultural adaptation were followed [22]. The scale was translated from its original language, English, into Turkish, then back-translated from Turkish to the original language, and finally back-translated into Turkish. First, professionally educated academic language specialists who were proficient in both English and Turkish translated the scale from its English form into Turkish. The scale was reduced to a single form after being translated into Turkish. A language specialist was given the completed Turkish form and requested that it be translated into English again. After reverse translation, the scale was translated back into Turkish by an academic in the psychology department who is proficient in English. Afterward, Turkish forms of the scale were assessed by experts in the fields of psychology and linguistics.

### Data analysis

IBM SPSS 26 was used to process the data. IBM SPSS Amos was subsequently used to analyze the scale to examine the validity of the study. The Cronbach’s alpha coefficient was used in the reliability study.

## Results

### Normality test

Before building the model and examining the fit indices, the observations were tested for compliance with the asssumption of normal distribution. Observations need to originate from a normal population that is both continuous and multivariate. However, data normality is rare in the reality of the real world [23]. Therefore, researchers use an estimation technique based on the Skewness and Kurtosis of the available data [24]. Maximum Likelihood (ML) approach technique is used for parameter estimation if the research variables are consistent with normal distribution. Skewness and Kurtosis values were examined to evaluate the normality assumption for the structural equation model. According to Tabachnick et al. [25], if the values of Skewness and Kurtosis are between − 1.5 and + 1.5, it can be said that the normal distribution condition is provided. Field stated that the normality assumption is met when Skewness and Kurtosis values are between − 2 and + 2 [26]. It can be observed that they are between − 1.5 and + 1.5 examining the Skewness and Kurtosis values of the variables used in the and therefore, it can be reported that the normality assumption is met (Table 1).

**Table 1:** Normality of data distribution

Variables	Mean	Standard deviation	Skewness	Kurtosis
GHOST1	2.06	0.846	0.096	-1.166
GHOST2	2.44	0.872	0.331	0.053
GHOST3	2.13	0.960	0.484	-0.402
GHOST4	2.40	1.056	0.253	-0.642
GHOST5	1.81	0.939	1.135	0.948
GHOST6	2.19	1.091	0.695	-0.290
GHOST7	1.98	1.070	0.835	-0.094
GHOST8	1.69	0.882	1.228	1.172

### Factor analysis

The scale’s items were analyzed, and the corrected item-total correlation was determined. Every statement on the scale had a corrected item-total correlation value greater than the 0.30 limit value [27, 28,29]. The corrected item-total correlation findings were in the range of 0.417-0.710. The Cronbach’s alpha reliability coefficient of the scale was 0.869.

### Exploratory factor analysis

Exploratory factor analysis is used to test construct validity [30]. Exploratory factor analysis was applied to determine whether the single-factor structure of the GHOST questionnaire in the original English language was valid for Turkey (*n* = 200). Barlett’s sphericity test and the Kaiser–Meyer–Olkin (KMO) test were used to determine whether the data were appropriate for factor analysis. The result of the Kaiser–Meyer–Olkin (KMO) test was 0.867. The results of Bartlett’s sphericity test, which revealed statistical significance (χ2 = 660.638; df = 28, *p* = 0.0001), indicated that the data could be used for factor analysis. The factorization in the analysis was performed via maximum likelihood extraction with promax rotation as per the original English language version. It is advised that this method be used when the data deviates from the assumption of multivariate normality [31, 32]. Promax, the most popular oblique rotation method, was used for factor rotation [33]. Many extraction approaches exist including Kaiser’s criteria (eigenvalue > 1 rule) [34], the Scree test [35], and the cumulative percent of variance extracted. The rule of eigenvalues being greater than 1 was applied as a factor extraction method. The factor loads of the scale items are between 0.417 and 0.78, and the variance explained by all the items is 46.237% (Table 2).

**Table 2:** Results of exploratory factor analysis

*Items and description*	*Eigen Value*	*Communalities*		*Factor loading*	*Variance*
		*Initial*	*Extraction*		
GHOST6. Size olan ilgileri tutarsızdır (Bazen çok ilgili, bazen tamamen ilgisizdirler).		0.54	0.60	0.78	46.237
GHOST5. Sizinle olan iletişimlerinde her zaman “Meşgulüm” ifadesini kullanırlar.		0.56	0.57	0.76	
GHOST3. Mesajlara verdikleri cevaplar karmaşık ve belirsizdir.		0.54	0.56	0.75	
GHOST4. Onlarla iletişimde kalmakta herhangi bir sorun yaşadınız mı? (Örn. Tek kelimelik cevaplar, kafa karıştırıcı emojiler, kısa cevaplar)	4.204	0.50	0.54	0.73	
GHOST8. Sizinle buluşmaya hevesli değillerdir.		0.46	0.44	0.66	
GHOST2. Mesajlarınıza geç cevap verirler.		0.42	0.39	0.64	
GHOST7. Sizinle kişisel bilgilerini paylaşmazlar.		0.43	0.38	0.61	
GHOST1. Daha öncesinde haber verilmeden planlarınız iptal edildi mi ya da ekildiniz mi?		0.20	0.19	0.44	

Notes: Extraction Method: Maximum likelihood extraction with promax rotation

### Confirmatory factor analysis

**Fig. 1:**
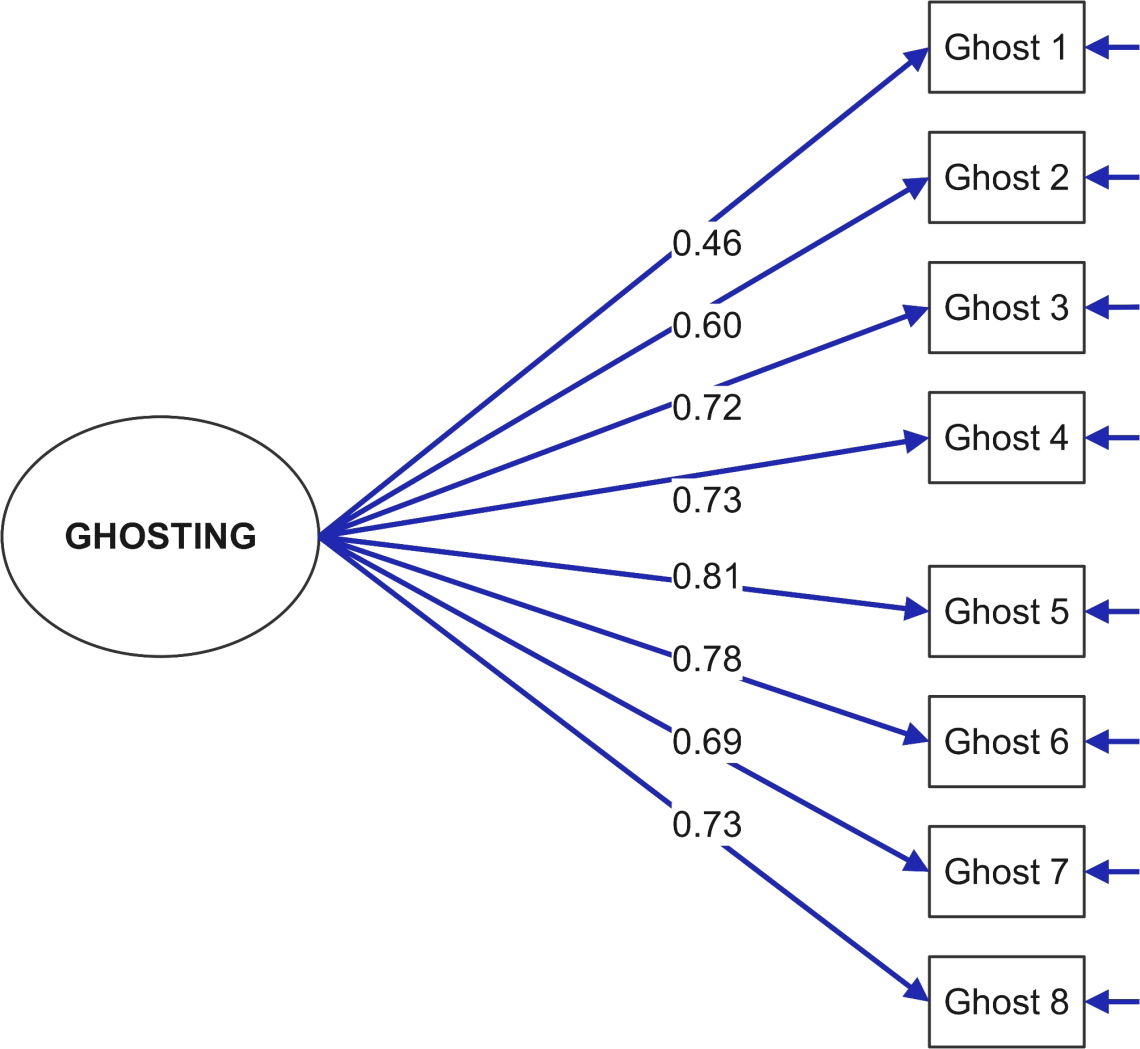
Path diagram of the confirmatory factor model

Fit indices for the confirmatory factor analysis were examined, and the *χ*^*2*^*/fd* ratio was found to be 1.284 (20.550/16 = 1.284). The following other fit indices were discovered: GFI = 0.97, CFI = 0.99, NFI = 0.97, RFI = 0.94, IFI = 0.99 and RMSEA = 0.03. Upon reviewing the goodness of fit results for the confirmatory factor analysis model, it is evident that the model exhibits concordance (Fig. 1).

## Discussion

Psychometric analyses of the Turkish version of the Ghosting Questionnaire are presented in this study. The Ghosting scale demonstrated sufficient construct validity and strong internal consistency. The original version of the 8-item Ghosting Questionnaire is comparable to that used in this study. The present findings reveal strong internal consistency, with a Cronbach’s α of 0.879 for the total scale, demonstrating comparability to, or even surpassing, the original English version (α = 0.74).

In the original scale development, there were initially 10 items. Each item had a heading that represented the following: Ghost 1 (Neglect), Ghost 2 (Avoidance), Ghost 3 (Lateness), Ghost 4 (Uncertainty), Ghost 5 (Inhibition), Ghost 6 (Barriers), Ghost 7 (Absence), Ghost 8 (Inconsistency), Ghost 9 (Vulnerability), and Ghost 10 (Withdrawal). However, as a result of the EFA, two items were removed from the scale (Ghost 2 and Ghost 5). The final version of the scale has 8 items in total. In the original scale validation study, Jahrami et al. [6] reported that the one-factor solution was appropriate for a sample with an average age of 26 years. The results of the factor analysis of the present study were also consistent with the original scale, which was found to have a single-factor structure.

In the recently published Urdu language adaptation study data by Husein et al. [36], 540 participants were reached. Cronbach’s alpha value was found to be 0.913, and the test-retest results with 100 participants were within the reliable range (ICC = 0.960; *p* < 0.001). According to the confirmatory factor analysis, a single-factor structure was observed, as in the original language and the current Turkish adaptation of the scale. The goodness of fit values of the data (Urdu version RMSEA = 0.045, CFI = 0.991, NFI = 0.983) were close to those of the Turkish adaptation (RMSEA = 0.03, CFI = 0.99, NFI = 0.97). Although the KMO values in the current study are higher than those in the other two studies (English: KMO = 0.914, Urdu: KMO = 0.918), they are within the validity limits (KMO = 0.876) [6, 27].

## Conclusion

In the modern environment, ghosting is a phenomenon that has become more widespread. Withdrawal occurs when someone abruptly and without cause breaks off all communication with another person. Social media, phone conversations, emails, and text messages can all be used for this purpose. The victim of ghosting may experience severe distress as well as confusion, disappointment, and rage as a result of the act. At its core, ghosting is an emotional manipulation tactic. An individual who ghosts another person manipulates their feelings by cutting off communication abruptly. Ghosting someone can be used as a form of punishment or as a way to escape a difficult situation. Ghosting victims may not always receive a justification or a conclusion.

The “Ghosting Questionnaire,” was translated into Turkish because of the growing interest in the topic. This has allowed researchers to make more progress and improved our understanding of ghosting behavior as a behavioral and psychological construct. By evaluating the dimensionality of the structure through EFA, it was found that the scale had a single-factor structure, as in the original model. CFA of the 8-item questionnaire was then conducted and showed a good fit, with all the items having sufficient factor loadings. The purpose of the Ghosting Questionnaire was to investigate the experience of ghosting, and it is a valid and reliable tool. The questionnaire was subjected to a psychometric analysis and was found to have adequate internal consistency, construct validity, and content validity to be used in future research on this contemporary phenomenon.

## Data Availability

Data associated with this paper can be produced on request from the first author.
